# The contribution of daytime sleepiness to impaired quality of life in NAFLD in an ethnically diverse population

**DOI:** 10.1038/s41598-022-08358-y

**Published:** 2022-03-24

**Authors:** Wenhao Li, Benjamin Karl Kadler, James Hallimond Brindley, Gillian Hood, Kalpana Devalia, John Loy, Wing-kin Syn, William Alazawi

**Affiliations:** 1 Barts Liver Centre, Blizard Institute, Queen Mary University of London, London, UK; 2 Bariatric Surgery Department, Homerton University Hospital, London, UK; 3 Division of Gastroenterology and Hepatology, Medical University of South Carolina, Charleston, SC USA; 4 Department of Physiology, Faculty of Medicine and Nursing, University of the Basque Country, Universidad del Pa S Vasco/Euskal Herriko Univertsitatea (UPV/EHU), Leioa, Spain; 5 Division of Gastroenterology and Hepatology, Saint Louis University School of Medicine, Missouri, USA

**Keywords:** Liver diseases, Non-alcoholic fatty liver disease, Non-alcoholic steatohepatitis, Diabetes, Obesity

## Abstract

Health-related quality of life (HRQoL) is lower in people with NAFLD compared to the general population. Sleep disturbance resulting in daytime sleepiness is common in patients with NAFLD, but the effect of daytime sleepiness on HRQoL in NAFLD is unclear. The prevalence and natural history of NAFLD vary in different ethnic groups, but there has been limited ethnic diversity in HrQoL studies to date. We aimed to assess whether daytime sleepiness is independently associated with reduced HRQoL in an ethnically diverse UK population. We conducted HRQoL assessments using SF-36 version 2 and Epworth Sleepiness Scale (ESS) questionnaires in 192 people with NAFLD. Multivariate linear regression was used to identify factors independently affecting HRQoL scales. People with NAFLD reported significantly reduced physical health-related SF-36 scores compared to the general UK population. South Asian NAFLD patients reported impairment in physical health, but not mental health, approximately a decade before White NAFLD patients. In multivariate linear regression, daytime sleepiness (ESS score > 10), was the most significant independent predictor of reduced physical health. Age, BMI and liver stiffness score were also significantly associated. HRQoL is impaired earlier in patients of South Asian ethnicity. ESS score > 10, indicative of excessive daytime sleepiness, is an independent predictor of reduced HRQoL in people with NAFLD regardless of ethnicity. Daytime sleepiness should be considered as a contributing factor to reduced HRQoL in clinical practice and when evaluating patient-related outcomes in clinical trials.

## Introduction

Non-alcoholic fatty liver disease (NAFLD) is the most common chronic liver disease worldwide affecting approximately 25% of the Western population and represents a spectrum of liver disease that ranges from benign steatosis to the inflammatory form, non-alcoholic steatohepatitis (NASH)^1^. Progression from benign steatosis to NASH can be associated with liver fibrosis, which can lead to cirrhosis, and significantly increases morbidity and mortality^2^.

Despite the growing awareness of the clinical outcomes associated with NAFLD, the wider impact of NAFLD from the patient’s perspective is not well understood. Health-related quality of life (HRQoL) assessments objectively examine the impact of a disease on an individual’s activities of daily living and allows for a common metric to compare to other diseases or to the general population. In addition to drug and disease related endpoints such as safety, tolerability and efficacy, changes in HRQoL have become important for regulatory bodies such as the European Medicines Agency (EMA) and the United States Food and Drug Administration (FDA) when evaluating new therapeutic agents^3^. A number of disease-specific HRQoL tools have been developed although the most widely-used tool is the Short Form-36 (SF-36) questionnaire, which consists of 36 questions related to physical and emotional function and general wellbeing^4^. Relevant to the current study, this questionnaire that was developed in the United States, has been validated in different populations globally, including United Kingdom^4^, India^5^, Pakistan^6^ and Bangladesh^7^.

A number of studies have reported reduced HRQoL in people with NAFLD compared to the general population, particularly in physical health-related domains, believed to be related to obesity and type 2 diabetes mellitus (T2DM)^8–10^ albeit other co-morbidites have received little attention**.** Although the prevalence and natural history of NAFLD varies in different ethnic groups^11^, there has been limited ethnic diversity in HrQoL studies to date. Taken together, we do not know whether patient factors such as ethnicity or other co-morbidities contribute to impaired HRQoL in NAFLD.

Daytime sleepiness can be caused by sleep deprivation, sedating medication and neurological disorders although another common cause is obstructive sleep apnoea (OSA); a breathing disorder characterised by recurrent collapse of the upper airway during sleep, leading to chronic intermittent hypoxia and itself can impair HRQoL. Obesity, OSA and NAFLD share a number of patient-related risk factors and putative disease mechanisms^12^. The prevalence and severity of OSA vary in different ethnic groups^13^ but whether this variation carries through to impact on HRQoL is unknown. Symptoms that are associated with OSA such as daytime sleepiness, snoring, fragmented sleep, fatigue and cognitive impairment can be difficult to elicit in routine clinical care. Polysomnography, the gold standard for diagnosing OSA, is not appropriate as a screening tool as it is labour intensive, time consuming and expensive. Therefore, OSA can remain undiagnosed, and so its impact on HRQoL in NAFLD remains unknown. Epworth Sleepiness Scale (ESS) is a validated screening questionnaire designed to identify individuals who have symptoms which can be consistent with OSA^14^ but this tool is not routinely utilised in HRQoL assessments in NAFLD hence the limited data in this space.

We hypothesise that ESS-determined symptoms consistent with OSA are independently associated with HRQoL impairment in people with NAFLD. We conducted SF-36 and ESS questionnaires at the same hospital visit in an ethnically diverse population of patients with NAFLD in order to understand the effect of ethnicity on these patient-related outcome measures and to determine factors associated with impaired HRQoL.

## Materials and methods

Patients over the age of 18 attending the hepatology outpatient clinics at Barts Health NHS Trust and for elective bariatric surgery at Homerton University Hospital Foundation Trust were invited to attend a further voluntary research visit, without financial incentive, for recruitment into non-commercial cross-sectional studies at Barts Liver Centre, Queen Mary University of London. Participants provided written informed consent and completed HRQoL assessments at enrolment. The studies were approved by the East London and City Regional Ethics Committee (reference numbers 18/LO/1759, 14/WA/1142) and performed in compliance with the Declaration of Helsinki. We included patients with evidence of liver steatosis, either by imaging (ultrasound, CT, MRI) or histology. Patients were excluded if they had any coexisting chronic liver disease diagnoses other than NAFLD, consumed alcohol greater than 14 units per week or had clinical features of decompensated cirrhosis. At enrolment, demographic data obtained included sex, age and ethnicity. Self-reported ethnicity was grouped into White, South Asian (Indian, Pakistani, Bangladeshi and Sri Lankan), Black or Other. Clinical and laboratory data obtained include body mass index (BMI), type 2 diabetes status, obstructive sleep apnoea diagnosis, glycated haemoglobin (HbA1c), drug history, platelet count, serum alanine aminotransferase (ALT) and aspartate aminotransferase (AST). Transient elastography was performed to measure liver stiffness for all recruited patients at enrolment according to standard clinical practice (reliable liver stiffness result based on successful reading rate > 60% and interquartile range of all readings < 30% of the median). Liver biopsy was performed in selected patients when clinically indicated or intra-operatively per-protocol at time of bariatric surgery. Each liver biopsy was reported by a single histopathologist (in routine clinical care) and were summarised according to the National Institutes of Health NASH clinical research network (Kleiner) criteria^15^.

Quality of life and daytime sleepiness assessments were conducted using Short Form-36 version 2 (SF-36v2) questionnaire and self-administered Epworth Sleepiness Scale (ESS) questionnaire. ESS scores were not performed in the bariatric cohort. SF-36 raw scores are combined to create scores for 8 sub-scales: physical functioning, role limitations due to physical health, emotional well-being, role limitations due to emotional problems, energy/fatigue, social functioning, pain, and general health. An overall physical health score (physical component scale [PCS]) and mental health score (mental component scale [MCS]) are derived from the sub-scale scores. PCS and MCS scores were compared against UK SF-36v2 normative data^4^. ESS questionnaire is a validated questionnaire used to assess daytime sleepiness, which consists of 8 questions scored from 0 to 3. ESS score greater than 10 is indicative of excessive daytime sleepiness, which may indicate OSA^14^.

### Statistical analysis

We compared demographic and clinical parameters with individual sub-scales, PCS and MCS of the SF-36v2 and ESS score in all patients. A separate analysis was performed in biopsy-proven patients to correlate quality of life indices with histological parameters. Continuous variables were expressed as mean and standard deviation or percentage. Categorical variables were expressed as percentage or proportion. Statistical analysis included Student’s t-test for comparison of two means for parametric data, the Mann–Whitney U test for comparison of two means for non-parametric data. Univariate and multivariate linear regression were performed to identify variables significantly associated with SF-36v2 PCS. Age, BMI, liver stiffness score, serum ALT, serum AST were analysed as continuous variables for univariate and multivariate analyses. All variables with *p* < 0.05 in the univariate analysis were included in multivariate analysis. Post hoc power calculations were performed based on group mean differences found in SF-36v2 PCS using G*Power (version 3.1.9.2). All other data handling and analyses were performed using GraphPad Prism (version 9.1.1), SPSS (version 26.0) and R (version 3.5).

### Ethics approval

The studies were approved by the local research ethics committees (reference numbers 18/LO/1759, 14/WA/1142) and performed in compliance with the Declaration of Helsinki.

### Consent to participate

All participants provided written informed consent at study enrolment.

### Consent to publication

All participants provided written informed consent at study enrolment.

## Results

A total of 266 patients were enrolled from January 2015 to January 2020, and after excluding 64 due to incomplete datasets, our cohort comprised 192 patients (Supplementary Table 1). All 192 patients completed an SF-36v2 questionnaire and 181 (94%) completed an ESS questionnaire. The mean age was 51.7 years, 42.2% were female (Table 1). Mean body mass index was 32.8 kg/m^2^, 47.4% had T2DM, mean liver stiffness measurement (LSM) was 10.2 kPa and 13.5% had known diagnosis of OSA. Mean ESS score (non-bariatric surgery cohort only) was 7.4 and 24.9% of patients had ESS score > 10. The mean Physical Component Summary (PCS) and Mental Component Summary (MCS) were statistically significantly lower than published scores from the general UK population^4^, with a greater effect seen in PCS (35.33 vs 50.02, *p* < 0.001) than in MCS (48.47 vs 50.05, *p* = 0.031) (Supplementary table 2).

**Table 1:** Clinical and Quality of Life-related factors in People with NAFLD from different ethnicities.

Variable	All (n = 192)		White (n = 83)		South Asian (n = 80)		Black (n = 14)		Other (n = 15)		White vs South Asian
	Mean	SD	Mean	SD	Mean	SD	Mean	SD	Mean	SD	*p* value
Age (years)	51.7	13.0	55.0	13.6	49.3	11.3	47.9	11.8	49.5	16.3	0.004
Gender (% female)	42.2%		41.0%		41.3%		35.7%		60.0%		0.999
BMI (kg/m^2^)	32.8	7.6	35.5	8.6	29.8	4.8	33.3	8.9	34.0	7.9	< 0.001
Liver stiffness score (kPa)	10.2	7.8	10.9	9.1	9.4	5.9	9.4	6.8	11.6	9.5	0.221
Obstructive sleep apnoea	13.5%		15.7%		8.8%		21.4%		20.0%		0.179
Type 2 diabetes	47.4%		37.3%		55.0%		28.6%		80.0%		0.022
HbA1c (mmol/mol)	57.1	13.6	57.6	15.9	57.9	13.2	60.0	18.4	49.0	5.4	0.938
ALT (U/L)	52.8	35.4	52.7	37.2	53.4	35.5	42.0	23.7	58.3	35.1	0.917
AST (U/L)	40.8	20.4	37.9	17.5	44.1	23.6	35.0	10.7	41.3	18.0	0.179
ESS Score	7.4	5.5	7.0	5.0	7.1	5.5	9.8	7.3	9.3	6.3	0.915
ESS score > 10	24.9%		22.4%		21.5%		41.7%		42.9%		0.999
**SF-36 scales**											
Physical Functioning	62.2	31.9	61.8	34.6	60.9	30.4	63.3	35.2	70.7	22.8	0.856
Role limitations due to physical health	59.1	45.0	56.3	46.4	61.1	44.4	64.6	44.5	58.9	45.6	0.509
Role limitations due to emotional health	68.9	43.4	72.8	42.1	63.7	44.7	83.3	33.3	64.3	49.7	0.194
Energy/fatigue	47.7	23.8	46.2	23.5	48.9	23.7	61.3	22.4	37.5	22.8	0.481
Emotional well-being	66.9	23.8	68.2	22.9	64.8	24.0	78.3	21.3	62.0	27.7	0.369
Social functioning	70.3	30.5	68.4	33.6	70.9	29.1	71.9	26.2	75.9	24.3	0.626
Pain	54.1	28.4	52.5	29.6	55.3	27.2	50.0	36.7	60.0	20.4	0.538
General health	48.3	23.5	49.1	25.8	47.2	20.2	60.4	29.0	39.3	20.0	0.594
Physical component scale (PCS)	35.3	14.8	34.4	16.5	35.9	13.3	34.2	16.7	38.4	11.8	0.534
Mental component scale (MCS)	48.5	13.4	49.3	13.4	47.3	13.3	55.8	8.6	44.2	15.1	0.363

Values are mean or %.

*ALT* alanine aminotransferase, *AST* aspartate aminotransferase, *BMI* body mass index, *ESS* Epworth Sleepiness Scale, *HbA1c* Haemoglobin A1c, *SD* standard deviation, *SF-36* 36-Item Short Form Survey.

Compared to patients with ESS ≤ 10, those with ESS score > 10 had a higher BMI (36.7 kg/m^2^ vs 31.3 kg/m^2^, *p* < 0.0001), higher prevalence of T2DM (58.9% vs 42.7%, *p* = 0.044), with lower PCS (26.9 vs 38.2, *p* < 0.0001) and MCS (39.3 vs 51.5, *p* < 0.0001) (Table 2). No differences in age, gender or ethnicity were observed. Similar proportions of patients reported use of medications with potential somnolent profiles (beta blockers, opioids, antihistamines, antidepressants) in both groups (14.3% vs 17.8%, *p* = 0.672). The chronicity of medication use was not recorded. Liver histology was available for 40 patients (Supplementary table 1) and no significant correlations between ESS score > 10 and histological parameters of NAFLD were identified (Supplementary table 3) in this group. 26.7% of patients with ESS score > 10 had non-obese BMI (adjusted for South Asian ethnicity^16^).

**Table 2:** Clinical and Quality of Life-related factors in People with NAFLD with low and high Epworth Sleepiness Scale (ESS) scores.

Variable	ESS score ≤ 10 (n = 136)		ESS score > 10 (n = 45)		*p* value
	Mean	SD	Mean	SD	
Age (years)	51.3	14.1	52.7	9.9	0.488
Gender (% female)	41.2%		44.6%		0.612
**Ethnicity**					
White	43.4%		42.9%		0.999
South Asian	45.6%		32.1%		0.075
Black	5.1%		12.5%		0.091
Other	5.9%		12.5%		0.102
BMI (kg/m^2^)	31.3	5.8	36.7	9.9	< 0.001
Liver stiffness score (kPa)	9.6	8.0	11.6	7.3	0.100
Obstructive sleep apnoea	7.4%		28.6%		< 0.001
Type 2 diabetes	42.6%		58.9%		0.044
HbA1c (mmol/mol)	58.0	14.0	54.6	12.3	0.435
ALT (U/L)	56.9	37.1	43.0	29.1	0.036
AST (U/L)	42.5	22.0	35.8	13.9	0.178
**SF-36 scales**					
Physical Functioning	67.6	29.8	46.0	33.0	< 0.001
Role limitations due to physical health	67.1	43.0	35.0	42.8	< 0.001
Role limitations due to emotional health	76.7	40.0	45.2	45.0	< 0.001
Energy/Fatigue	53.7	22.1	29.4	19.0	< 0.001
Emotional well-being	72.6	21.1	49.8	23.2	< 0.001
Social functioning	77.0	27.7	50.0	29.7	< 0.001
Pain	61.0	25.6	33.3	26.2	< 0.001
General health	54.0	21.9	30.8	19.4	< 0.001
Physical component scale (PCS)	38.2	13.4	26.9	15.5	< 0.001
Mental component scale (MCS)	51.5	12.0	39.3	13.2	< 0.001

Values are mean or %.

*ALT* alanine aminotransferase, *AST* aspartate aminotransferase, *BMI* body mass index, *ESS* Epworth Sleepiness Scale, *HbA1c* Haemoglobin A1c, *SD* standard deviation, *SF-36* 36-Item Short Form Survey.

**Figure 1:**
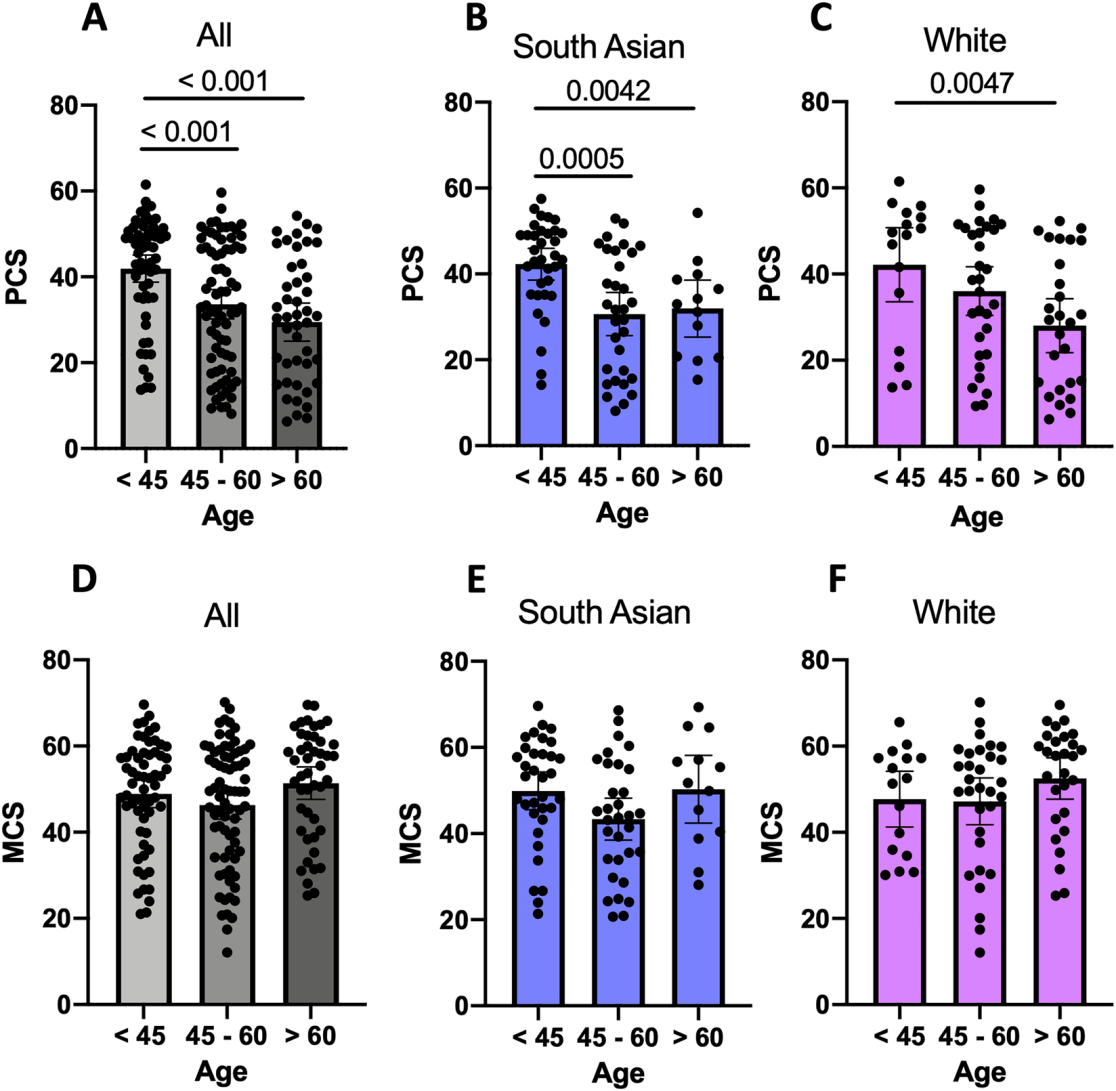
Physical and Mental Component Scores stratified by age. *PCS* physical component summary, *MCS* mental component summary. (**A**) All NAFLD PCS stratified by age; (**B**) South Asian PCS stratified by age; (**C**) White PCS stratified by age; (**D**) All NAFLD MCS stratified by age; (**E**) South Asian MCS stratified by age; (**F**) White MCS stratified by age.

**Figure 2:**
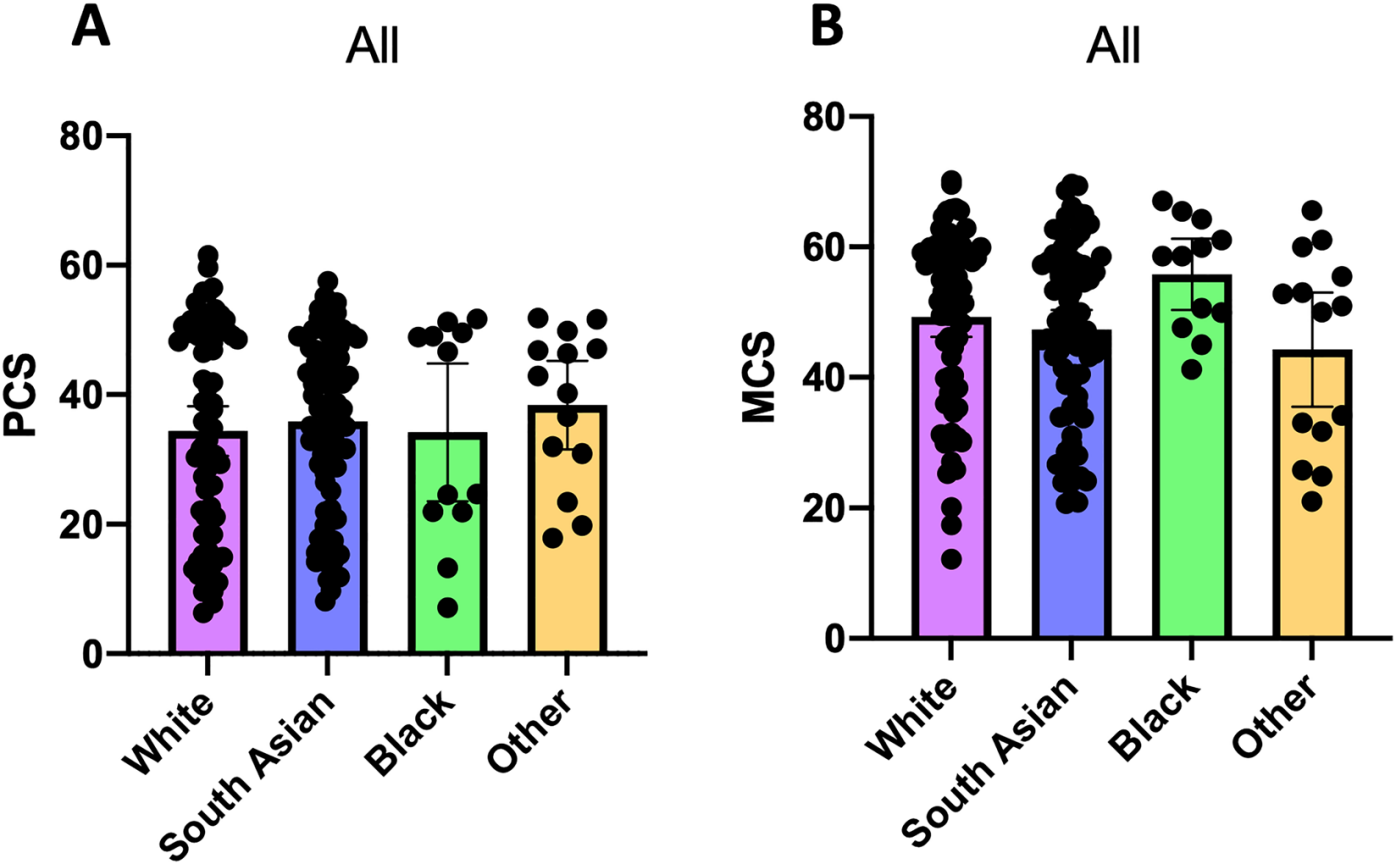
Physical and Mental Component Scores stratified by ethnicity. *PCS* physical component summary, *MCS* mental component summary. (**A**) All NAFLD PCS stratified by ethnicity; (**B**) All NAFLD MCS stratified by ethnicity.

The two largest ethnic groups were White (n = 83, 43.2%) and South Asian (n = 80, 41.7%). Compared to White patients, South Asian patients were younger (49.3y vs 55.0y, *p* = 0.004), with lower mean BMI (29.8 kg/m^2^ vs 35.5 kg/m^2^, *p* < 0.001), had a higher prevalence of T2DM (55.5% vs 37.4%, *p* = 0.022), albeit with comparable LSM scores (9.4 kPa vs 10.9 kPa, *p* = 0.221), ESS scores (7.0 vs 7.1, *p* = 0.915), serum levels of ALT (53.4 IU/ml vs 52.7 IU/ml, *p* = 0.917) and AST (44.1 IU/ml vs 37.9 IU/ml, *p* = 0.179) (Table 1). PCS, but not MCS, reduced with advancing age (Fig. 1A,D). We found a reduction in PCS, but not MCS, at an earlier age (by approximately 9 years) in patients of South Asian ethnicity compared to White ethnicity (Fig. 1B,C,E,F, Supplementary Table 4). PCS was lower in the 45–60 age group compared to under 45 age group in South Asian patients, but in patients of White ethnicity, a significant decline in PCS only occurred in patients aged over 60. There were no differences in PCS and MCS when White and South Asian groups of all ages were compared (Fig. 2A,B). Across all ethnicities, the presence of T2DM was associated with lower scores in role limitations due to physical health, general health and PCS compared to people with NAFLD who did not have diabetes (49.4 vs 67.5, *p* = 0.007; 42.5 vs 53.2, *p* = 0.002; 32.5 vs 37.8, *p* = 0.016 respectively) (Supplementary Table 2).

In univariate linear regression, age, BMI, diabetes diagnosis, liver stiffness score, ESS score > 10 and OSA diagnosis were negatively associated with PCS (Table 3). OSA and diabetes diagnoses no longer had significant associations with PCS on multivariate linear regression, but the other variables remained statistically significant. ESS score > 10 was the most significant independent predictor of PCS. Sensitivity analyses showed that after firstly excluding those with known diagnosis of OSA (n = 26) (Supplementary Table 5); secondly excluding those with use of medications with potential somnolent profiles and known diagnosis of OSA (n = 48), ESS score > 10 remained the strongest independent predictor of PCS (Supplementary Table 6). Post hoc power analysis determined the total study size of 192 was adequately powered (1 − β = 0.997) to detect statistically significant differences PCS between ESS ≤ 10 v and ESS > 10 groups (Supplementary Table 7).

**Table 3:** Factors Associated with Physical Component Summary (PCS) of SF-36 in all People with NAFLD by Univariate and Multivariate Linear Regression (n = 177).

Variables	Univariate analysis			Multivariate analysis		
	B coefficient	(95% CI)	*p* value	B coefficient	(95% CI)	*p* value
Age (years)	− 0.322	(− 0.479 to − 0.164)	< 0.001	− 0.252	(− 0.400 to − 0.104)	0.001
Gender (male)	2.495	(− 1.934 to 6.924)	0.268			
Ethnicity (White)	− 1.644	(− 6.053 to 2.765)	0.463			
Ethnicity (South Asian)	0.965	(− 3.420 to 5.350)	0.665			
BMI (kg/m^2^)	− 0.780	(− 1.086 to − 0.475)	< 0.001	− 0.504	(− 0.814 to − 0.195)	0.002
Liver stiffness score (kPa)	− 0.626	(− 0.883 to − 0.370)	< 0.001	− 0.318	(− 0.580 to − 0.056)	0.018
Type 2 diabetes	− 5.297	(− 9.593 to − 1.000)	0.016	− 1.286	(− 5.375 to 2.803)	0.536
Obstructive sleep apnoea	− 7.358	(− 13.915 to − 0.8)	0.028	− 0.948	(− 6.915 to 5.020)	0.754
ALT (U/L)	0.032	(− 0.044 to 0.107)	0.407			
AST (U/L)	0.108	(− 0.047 to 0.263)	0.168			
ESS score > 10	− 11.298	(− 16.040 to − 6.556)	< 0.001	− 8.329	(− 12.868 to − 3.790)	< 0.001

*ALT* alanine aminotransferase, *AST* aspartate aminotransferase, *BMI* body mass index, *ESS* Epworth Sleepiness Scale.

## Discussion

This study, conducted in an ethnically diverse urban population showed that high Epworth Sleepiness Score, indicative of excessive daytime sleepiness, is a significant independent predictor of impaired HRQoL in patients with NAFLD. Overall, people with NAFLD had lower quality of life (particularly physical health scores) compared to the general population, in keeping with previous reports^8–10^. We found that ethnicity is associated with the age of onset of decline in physical health scores in people with NAFLD which was significantly earlier in patients of South Asian compared to White ethnicity. However, no significant differences in OSA prevalence or ESS scores were observed.

Other than NAFLD, previous studies have demonstrated variability in the prevalence of daytime sleepiness and OSA in different ethnicities, associated with a range of conditions including obesity, type 2 diabetes, cardiovascular disease and depression. In an atherosclerosis study of 2230 participants, higher prevalence of daytime sleepiness and undiagnosed OSA were reported in those of Black, Hispanic and Chinese ethnicities compared to White ethnicity^17^. The National Health and Wellness Survey from 7239 diabetic patients found the strongest predictors of sleep disturbance and daytime sleepiness were obesity, White ethnicity, female gender, low income, and smoking^18^. Longitudinal analysis of 41,094 participants of the UK Biobank found obesity, ethnicity (Black, Asian and mixed), depression, and social deprivation were the main factors associated with poor sleep and associated symptoms^19^. In Asian populations, daytime sleepiness and OSA were associated with subclinical atherosclerosis and diabetes irrespective of BMI^20,21^. The variability in these observations are not fully understood, but are likely to be multifactorial, associated with genetic, biological, social and environmental factors. Recent genetic studies have identified methylation sites in multiple genes specifically associated with daytime sleepiness in African Americans^22^. Differences in craniofacial structures have been linked to elevated risk of OSA and associated symptoms despite lower rates of obesity in Asian populations compared to White and Black ethnicities^23,24^.

In our study, 13.5% of participants had a diagnosis of OSA at enrolment, but a significantly higher proportion (24.9%) recorded ESS scores > 10. To our knowledge, this is the first report that shows ESS score > 10 is a significant independent predictor of physical HRQoL, in addition to factors known to be associated with impaired physical health scores in NAFLD; older age, female gender, BMI, presence of T2DM, degree of fibrosis including cirrhosis^8–10,25–27^. In our study, ESS score > 10 was associated with metabolic co-morbidites (obesity, T2DM), and liver stiffness score by transient elastography. Our study was not designed to detect associations of HRQoL or sleepiness with histology and the lack of statistical associations may be related to the low proportion of patients in our study who had liver biopsy as part of routine clinical care (21%). Nevertheless, our findings were congruent with findings by Newton et al.^28^, which reported perceived fatigue experienced by patients with NAFLD was associated with daytime sleepiness, but not liver disease severity. However, a Swiss study of sleep disturbance in patients with biopsy-proven NAFLD, insulin resistance and elevated serum transaminase levels^29^ showed an association between sleepiness and NASH on biopsy albeit the Swiss cohort differed in that patients had lower mean BMI, ESS and fibrosis scores, and higher mean ALT. Continuous positive pressure ventilation (CPAP) is the primary treatment for OSA, associated with improvements in OSA symptoms, HRQoL^30^ and reduction in ESS scores^31^. However, a randomised controlled trial of CPAP, for 6 months in people with NAFLD and OSA did not demonstrate regression of hepatic steatosis or fibrosis^32^ suggesting that mechanisms beyond hypoxaemia and fibrosis regression influence HRQoL in patients with NAFLD and OSA. In addition, further work is needed to assess the impact of daytime sleepiness on key lifestyle determinants, such as exercise capacity, mood, appetite, eating patterns and other activities or daily living, which may reduce the ability of people with NAFLD to make substantive lifestyle changes. Daytime sleepiness in people with NAFLD without obesity was high in our study (26.7% of all participants with ESS score > 10 did not have obesity), in keeping with similar findings in a non-morbidly obese Italian NAFLD cohort^33^. Future studies with polysomnography to confirm OSA are warranted in this subpopulation of NAFLD with high ESS scores.

To date, the majority of studies assessing HRQoL in NAFLD have been conducted in the United States with preponderance of White ethnicity (> 73%)^8–10,26,27^. In patients with advanced NASH fibrosis enrolled in the multinational phase 3 STELLAR trials of selonsertib, there were significant differences in HRQoL scores between White, Asian and Black groups of participants, although only 1.5% of the total cohort were of Black ethnicities^9^. We previously reported that patients of South Asian ethnicity have a greater risk of developing NAFLD at a younger age (by approximately a decade), with a lower BMI compared to White patients with comparable disease stages^34^. This is mirrored in our current study by the reduction in physical health scores despite lower BMI, and together, this signifies that South Asian patients with NAFLD suffer physical impairment from a much younger age compared to White patients. The underlying causes for this are likely to be multifactorial, though higher rates of diabetes were observed in South Asian people and Chawla et al. reported a significant reduction in physical health scores in people with NASH and diabetes compared to non-diabetic NASH patients^25^. Further work is required to understand the effect of South Asian ethnicity on the natural history of NAFLD and why it impacts HRQoL at younger ages. While higher rates of diabetes may play a role, other factors may include diet, genetics, cultural health behaviours and social deprivation, which were not explored in this study. Our collective work highlights the importance of ethnicity when evaluating NAFLD severity from a clinician as well as patient perspective.

This study has both strengths and limitations. Firstly, the patients enrolled reflect real world NAFLD in clinical practice, not restricted by stringent clinical trial criteria. Secondly, our study population is more ethnically diverse compared to previous studies, with significant proportion of South Asian patients. Thirdly, SF-36v2 questionnaire was used to evaluate HRQoL in this study. Although this questionnaire is not specifically designed to evaluate HRQoL in chronic liver disease, unlike the Chronic Liver Disease Questionnaire (CLDQ), it is a widely used HRQoL assessment tool validated in different populations globally that allows for comparisons with different disease states and the general population, which is not possible with CLDQ. Furthermore CLDQ was developed and validated using SF-36 as the gold standard^25^. ESS alone has modest discriminatory ability as a screening tool for OSA (sensitivity ranging from 38 to 66% and specificity ranging from 48 to 79% with ESS score > 10 as threshold)^35,36^. Although the focus of our study was to elicit the symptoms of daytime sleepiness rather than to formally diagnose OSA, one limitation of our study is that polysomnography was not used to confirm those suspected of OSA based on the ESS scores. High ESS score reflects a state of fatigue, which is also 1 of 8 major components measured in SF-36 questionnaire. A limitation in our study is the absence of control group(s) to assess whether liver disease or other associated factors contribute to fatigue in patients with NAFLD, but again, this was not an aim of our study. Finally, the cross-sectional nature of this study prevents causal conclusions to be drawn from the reported correlations. Very few longitudinal HRQoL studies have been conducted to date in NAFLD. The PIVENS trial for NASH reported no significant changes in SF-36 scores between the study arms after 96 weeks^37^. In patients with NASH F2 – 3 fibrosis treated for 24 weeks with selonsertib in a phase 2 clinical trial, patients in whom there was improvement in a surrogate marker of fibrosis (percentage collagen) but not histological NAFLD activity score also recorded improvement in physical health scores^38^. Future prospective longitudinal studies are warranted to investigate strategies that can improve physical impairment in people with NAFLD of different ethnicities.

## Conclusion

In this study, we demonstrate that people with NAFLD from an ethnically diverse UK population report lower quality of life compared to the general population. Physical health in particular is significantly reduced. Our work also highlights the importance of ethnicity in HRQoL outcomes in NAFLD. South Asian people with NAFLD report physical health impairments from a significantly younger age compared to White people with NAFLD despite more favourable BMI. We identify undiagnosed daytime sleepiness as a major component in NAFLD HRQoL which should be considered in clinical practice and when evaluating patient-related outcomes in clinical trials through the use of tools such as the Epworth Sleepiness Scale.

## Supplementary Information

Supplementary Information.

## Data Availability

All anonymised data available by request directly to corresponding author.
