# Supplementary material for: The contribution of daytime sleepiness to impaired quality of life in NAFLD in an ethnically diverse population
Source: Sci Rep. 2022 Mar 24;12:5123. doi: 10.1038/s41598-022-08358-y (PMC8948283; doi:10.1038/s41598-022-08358-y)
Supplement: Supplementary file 1 — Supplementary Information. [file 41598_2022_8358_MOESM1_ESM.docx]

**The contribution of daytime sleepiness to impaired quality of life in NAFLD in an ethnically diverse population**

Wenhao Li ^1^, Benjamin Karl Kadler ^1^, James Hallimond Brindley ^1^, Gillian Hood^1^, Kalpana Devalia ^2^, John Loy ^2^, Wing-kin Syn^3 4^, William Alazawi ^1^

**Supplementary data**

**Supplementary Table 1 –** Clinical and Quality of Life-related factors in biopsy proven NAFLD patients (n = 40)

**Supplementary Table 2** – SF-36 component scores in NAFLD patients with and without diabetes compared to the general UK population

**Supplementary Table 3** – Pearson correlations (Epworth Sleepiness Scale score > 10 compared to liver histology parameters, n = 30)

**Supplementary Table 4** – Age comparison of White and South Asian NAFLD patients with impaired physical component summary (PCS) and mental component summary (MCS) scores (Impairment defined as PCS and MCS scores below the 25^th^ percentile)

**Supplementary Table 5** – Sensitivity analysis of factors associated with physical component summary (PCS) of SF-36v2 in the NAFLD patients after excluding NAFLD patients with known obstructive sleep apnoea (n = 166)

**Supplementary Table 6** – Sensitivity analysis of factors associated with physical component summary (PCS) of SF-36v2 in the NAFLD patients after excluding NAFLD patients with known obstructive sleep apnoea and taking reported use of medications with potential somnolent side effects (n = 144)

**Supplementary Table 7**  – Post hoc power analysis of study

**Supplementary Table 1 –** Clinical and Quality of Life-related factors in biopsy proven NAFLD patients (n = 40)

**Supplementary Table 2** – SF-36 component scores in NAFLD patients with and without diabetes compared to the general UK population

**Supplementary Table 3** – Pearson correlations (Epworth Sleepiness Scale score > 10 compared to liver histology parameters, n = 30)

**Supplementary Table 4** – Age comparison of White and South Asian NAFLD patients with impaired physical component summary (PCS) and mental component summary (MCS) scores (Impairment defined as PCS and MCS scores below the 25^th^ percentile)

**Supplementary Table 5** – Sensitivity analysis of factors associated with physical component summary (PCS) of SF-36v2 in the NAFLD patients after excluding NAFLD patients with known obstructive sleep apnoea (n = 166)

**Supplementary Table 6** – Sensitivity analysis of factors associated with physical component summary (PCS) of SF-36v2 in the NAFLD patients after excluding NAFLD patients with known obstructive sleep apnoea and taking reported use of medications with potential somnolent side effects (n = 144)

**Supplementary Table 7** – Post hoc power analysis of study

| Effect size d = 0.807 | ESS ≤ 10 sample size = 136 |
| --- | --- |
| α = 0.05 | ESS > 10 sample size = 45 |
| **Power = 0.997** | |
